# Out-of-School Learning in the Wadden Sea: The Influence of a Mudflat Hiking Tour on the Environmental Attitudes and Environmental Knowledge of Secondary School Students

**DOI:** 10.3390/ijerph20010403

**Published:** 2022-12-27

**Authors:** Till Schmäing, Norbert Grotjohann

**Affiliations:** Didactics of Biology (Botany/Cell Biology), Faculty of Biology, University of Bielefeld, 33615 Bielefeld, Germany

**Keywords:** UNESCO World Heritage Site, Wadden Sea, out-of-school learning, environmental education, environmental attitudes, environmental knowledge

## Abstract

In this study, the Wadden Sea, as an out-of-school learning site, is considered from an empirical-research perspective of environmental education. The Wadden Sea in Germany is part of the largest contiguous mudflat area in the world. Although much evidence is already available on different teaching and learning processes at various out-of-school learning sites, this is not yet the case for the Wadden Sea. This research gap was taken up. In this paper, 427 students (mean age: 11.74 years) participated in a mudflat hiking tour. A pre–post-test design followed by a retention test was used to determine the impact of this on participants’ environmental attitudes and environmental knowledge. The single factor analyses of variance with repeated measures demonstrated that the mudflat hiking tour had both a positive short-term and a positive long-term impact on environmental attitudes, as well as on environmental knowledge. All three constructs could, therefore, be positively influenced by the intervention. Correlation analyses revealed a positive relationship between environmental attitudes and environmental knowledge at three measurement time points. These results empirically confirm the potential of the Wadden Sea as an out-of-school learning site for environmental education with students from secondary schools. The effectiveness of non-formal education in this ecological environment can be proven.

## 1. Introduction

Out-of-school learning sites offer great potential and should be used more frequently, especially in science education [1]. Among other things, out-of-school learning promotes children’s and adolescents’ independence, creativity, self-organization skills, and forward-looking, reflective, networked, and interdisciplinary thinking, and it counteracts their increasing alienation from nature [2]. For this very reason, out-of-school teaching and learning processes are highly relevant for environmental and biology education. The literature shows that out-of-school experiences with nature positively correlate with an interest in biology [3]. To foster this interest in learning about biology, and, also, about the living environment in general, out-of-school experiences should be given a higher weighting [4]. Moreover, it is important to explore processes at out-of-school learning sites so that the results obtained from this empirical research can contribute to the optimization of appropriate educational interventions. In environmental education, there are several studies that look at different out-of-school learning sites. A selection of key findings at different out-of-school learning sites is presented below as examples.

There is already a wealth of research findings in botanical gardens as an out-of-school learning site. Thus, they can be an educational site that vividly conveys the plant world, its endangerment, and the efforts for the conservation of plant biodiversity [5]. Students can achieve short-term and long-term knowledge acquisition in botanical gardens with a thematic environmental education program [6], and their environmental attitudes can also be positively influenced [7]. In addition, the high motivation among students during out-of-school learning at this learning site has been demonstrated [8]. Nevertheless, in the context of botanical gardens, the use of suitable educational material should be considered [9].

Another learning site that has already been empirically considered from the perspective of environmental education is the zoo. At this point, it should be emphasized that—as with botanical gardens—the selected studies look at a specific educational intervention at a specific learning site so that the findings cannot automatically be transferred to other zoos or botanical gardens nor the interventions implemented there. Nonetheless, it can be argued that what emerges from the research are the multifaceted opportunities for high-quality environmental education in zoos. For example, it is possible to have a short-term and a long-term positive impact on knowledge through out-of-school learning processes in zoological institutions [10,11]. Connectedness with nature can also be promoted with a visit to the zoo [12,13]. Again, it must be stated that there are differences in terms of the effects of the different concrete activities. For example, negative changes in understanding of animals and their habitats occur more frequently during unguided visits than during guided visits [14].

The two learning sites taken up as examples have in common that they are facilities that are primarily oriented towards human exploration. However, out-of-school learning, especially in environmental education, can also take place in natural environments. One ecosystem that is frequently explored with school classes is the forest. In Germany, the so-called *Waldjugendspiele* [15] have existed for more than 50 years. The overall objective of these programs is to counteract the alienation from nature and to familiarize the students with the forest as a habitat, using all their senses and having fun [16]. Various methods are used, including an animal quiz [17]. These events are also used to implement environmental education research (e.g., [18]).

Another ecosystem of great importance in Germany is the Wadden Sea. Despite its great potential for out-of-school learning in environmental education [19] as well as its international significance as a UNESCO World Heritage Site and the associated special suitability as a learning site [20], it has hardly been considered from an empirical-research perspective [21,22]. The present study addresses this research gap and determines the effects of an exploration of this ecosystem by secondary school students. The potential influences of this field trip on the participants’ environmental attitudes and environmental knowledge will be considered. As can be seen from the studies cited so far, both constructs are relevant to the domain of out-of-school learning in environmental education.

The environmental attitudes are described from a theoretical point of view regarding preservation and utilization [23]. The first factor represents the preference for protecting nature; the second factor describes the preference for exploiting nature [24]. Both factors are addressed in this study, and the changes resulting from mudflat hiking tour are examined. The same applies to environmental knowledge about the Wadden Sea ecosystem. This is described according to Kuckartz [25] with a particularly pronounced subject-specific component in the present study and can, thus, be named as system knowledge [26]. This is the basis for the development of further forms of knowledge [27] and should, therefore, be promoted especially among young people [28]. The theoretical references to both environmental attitudes and environmental knowledge will be deepened in the description of the measurement instruments used.

## 2. Methods

In this section the implemented methodological procedure is described in detail. For this purpose, the concrete questions are derived and formulated at the beginning, and the pedagogical intervention carried out with the students in the Wadden Sea is vividly presented with reference to photos. Based on this, the sample is described, the measuring instruments used are discussed, and the study design is presented. This is followed by notes on the statistical analysis procedures used.

### 2.1. Research Questions

There are many ways to conduct research at an out-of-school learning site and examine the effects of the learning and teaching that takes place there. In the present study, the research design was based on other scientific studies. Orientation in terms of content also followed in this way. Thus, two constructs were selected that were also relevant in the studies outlined in the introduction.

The UNESCO World Heritage Site Wadden Sea is, as will be seen in the next section, ideal to implement environmental education/education for sustainable development. In this context, it makes sense to investigate the influence of a field trip to the Wadden Sea on the environmental attitudes of the students. Therefore, the first research question is formulated:

1. Does a mudflat hiking tour have an influence on the environmental attitudes of the participants?

In addition to the construct of environmental attitudes, which is an affective component, the study also considers a cognitive level with the potential acquisition of environmental knowledge. The second research question is:

2. Does a mudflat hiking tour have an impact on participants’ environmental knowledge of this ecosystem?

Since all participants were asked about both their environmental attitudes and their environmental knowledge, a potential relationship between these two constructs will be investigated. Therefore, this aspect is investigated with the third question:

3. Is there a correlation between environmental attitudes and environmental knowledge about the Wadden Sea?

### 2.2. Intervention

**Figure 1:**
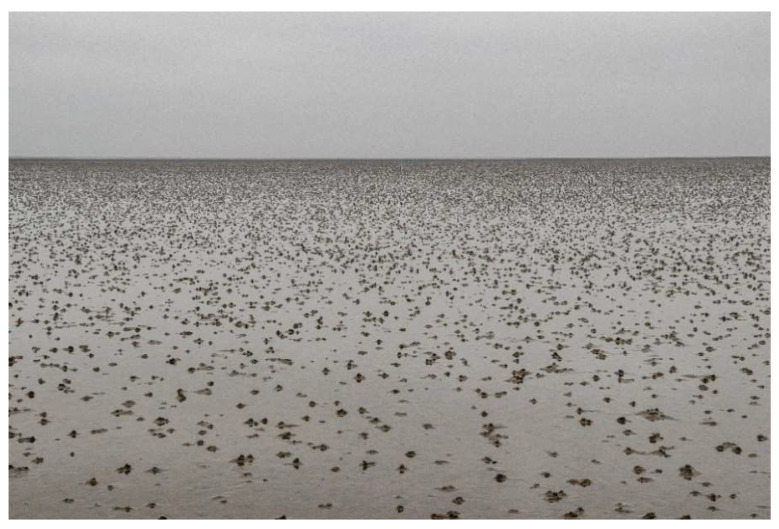
The Wadden Sea. At first glance, only a large area of mud and the heaps of lugworms are visible. On the mudflat hiking tour, the students experienced the many zoological and botanical characteristics of the ecosystem.

As outlined above, secondary school students participating in this study took part in a field trip to the Wadden Sea, located on the German North Sea coast. The first areas of the Wadden Sea were declared a UNESCO World Heritage Site in 2009 [29], so it is a recommended learning site as a World Heritage Site [20]. The Wadden Sea of the North Sea extends along the coasts of Denmark, Germany, and the Netherlands [30]. It has a length of about 450 kilometers [31] and an area of about 13,500 km^2^ [32]. It is the largest contiguous mudflat area in the entire world [33]. To some, the Wadden Sea may, at first glance, look like a lifeless expanse of mud (Figure 1). Nevertheless, more than 10,000 different species live in this ecosystem [34].

The Wadden Sea can be accessed around the time of low tide, as the tidal flats are not covered by water during this period. In the present study, participants took part in a guided tour of the Wadden Sea lasting about two hours. This involves exploring the ecosystem together with a mudflat guide in groups. This person carried a digging fork, scoops, and sieves. With these instruments, the participants were able to carefully take some living creatures from the ground and the puddles. Afterwards, these could be observed. The mudflat guide also took along pointing cards to demonstrate and explain the way of life of the individual animals with scientific illustrations. The group walked in the Wadden Sea and stopped at different places. At these stops, the participants formed a small circle and were informed by the mudflat guide about the procedure at this station. In the area of each circle, the students explored the Wadden Sea and its creatures with the mentioned instruments or simply made observations (for example, of traces or molting). The excursion to the Wadden Sea is an integral part of the educational concept of the national park facilities of the Lower Saxony Wadden Sea National Park [35]. Out-of-school learning is also relevant in the legal requirements for biology education [36,37]. The conducted mudflat hiking tour allowed students to explore the ecosystem on site. In general, the mudflats were entered barefoot so that a special experience could be ensured. During the mudflat tour, various animal and plant species were introduced in more detail as described. The next section describes key elements of the mudflat hiking tour in which the students participated.

**Figure 2:**
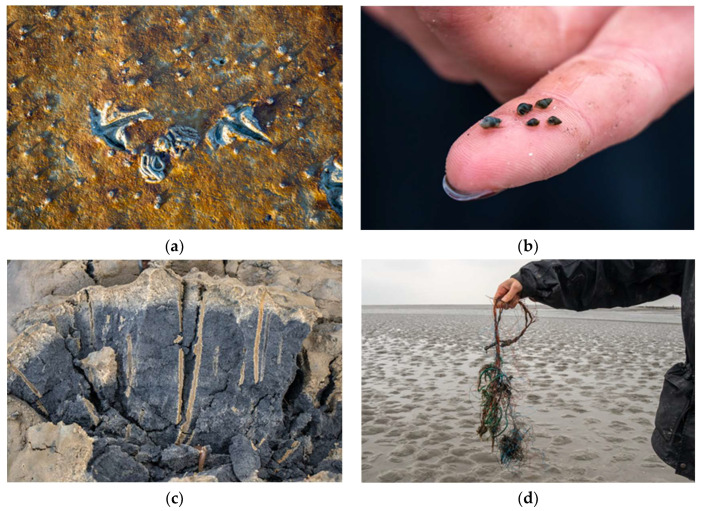
Impressions of the mudflat hiking tour. (**a**) shows the bottom of the tidal flats covered with diatoms. Also visible are traces of a bird, heaps of a lugworm, and laver spire shells. These can be seen on a finger dome in (**b**). (**c**) illustrates the tubes of worms, these are the light areas in the tidal flat bottom. The Wadden Sea is subject to various types of pollution, (**d**) shows plastic fishing nets. These were found in the Wadden Sea during the field trip.

Of particular importance for the ecosystem are the diatoms (*Bacillariophyta*), as these are primary producers [38]. These diatoms are recognizable as a kind of brown coating on the tidal flat bottom (Figure 2a), so they can be directly perceived by the participants. The diatoms are eaten by, for example, the laver spire shell (*Hydrobia ulvae*), of which there are up to 300 000 individuals per square meter [39]. During the mudflat hiking tours, participants carefully lifted the tiny snails from the mudflats with a sieve and were able to view them more closely (Figure 2b). In addition to the laver spire shells, students were able to observe and, in some cases, carefully touch other animal species. These included the common cockle (*Cerastoderma edule*), the common shore crab (*Carcinus maenas*), the common shrimp (*Crangon crangon*), the estuary ragworm (*Nereis diversicolor*), and the lugworm (*Arenicola marina*). The latter lives in a U-shaped living tube below the surface of the mudflat bottom and was carefully excavated by the mudflat guide with a fork. In Figure 2c, tunnels of a worm can be seen. The mudflat guide provided key information on all species, using cards to illustrate characteristic features or behaviors of the creatures. In addition, with a reference to education for sustainable development, attention was drawn throughout to the threats to individual species and the entire ecosystem. (Plastic) trash was found directly in the Wadden Sea, such as the remains of fishing nets shown in Figure 2d. The frequency of the finds is not surprising, as an average of 712 pieces of garbage can be found on a 100-meter stretch of beaches in the OSPAR region [40]. The Wadden Sea is also exposed to other threats including shipping from oil rigs or pipelines [41]. Ultimately, anthropogenic climate change brings challenges that can threaten the existence of the entire ecosystem [42]. All of these content areas were addressed throughout the mudflat hiking tour to help participants understand the relevance of environmentally friendly behaviors to protect this unique ecosystem. Nevertheless, it must be stated that the transfer of knowledge has taken on an overarching relevance in this intervention. Education for sustainable development is much more far-reaching than this. However, among other things, the biodiversity that can be experienced during the mudflat hiking tour can be highlighted as a key theme of education for sustainable development [43].

### 2.3. Participants

A total of 427 students from secondary schools participated in the study and explored the Wadden Sea UNESCO World Heritage Site accompanied by a mudflat guide. The average age of the respondents is 11.74 years (range: 10–17 years), and 56.46% of them are female. The students attend school forms that enable them to achieve the A-levels in the period of the upper school. All students joined a mudflat hiking tour with the same concept, which has been described in Section 2.2. So, the basic educational program was the same. Data collection began in August 2021 and ended in September 2022. The majority of excursions were conducted in the spring and summer of 2022.

### 2.4. Measuring Instruments

As explained with the formulation of the questions, two different constructs are considered in the study with environmental attitudes and environmental knowledge. The environmental attitudes are determined with the 2-Major Environmental Values (2-MEV) model according to Bogner and Wiseman [23]. As outlined in the introduction, there are two factors, Preservation and Utilization, which are opposite to each other. The factor preservation is characterized with the preference of nature conservation; the factor utilization with the preference of nature exploitation. This dichotomous approach derives from the development of this instrument and from the first studies in which it has been used [24,44,45]. By now, this measurement tool has been independently confirmed internationally several times [46,47,48,49,50] and has been used in many studies related to environmental education that have implemented an educational intervention [7,51,52]. Therefore, 2-MEV-model is predestined for use in the present study. Table 1 shows the scale used. The preservation and utilization factors are each represented by ten items.

For the determination of the environmental knowledge about the UNESCO World Heritage Wadden Sea, there was no connecting point in the literature that considers this specific ecosystem. Therefore, following other research [53,54,55] and recommendations from the literature [56,57], a knowledge test had to be designed together with different experts on the Wadden Sea in order to be able to survey the environmental knowledge about the Wadden Sea. The conception according to scientific criteria and a first application to investigate the effects of a teaching station on the Wadden Sea on environmental knowledge were successful [28] so that this instrument is also suitable for use in this study.

The environmental knowledge test includes ten items, which consider different contents about the Wadden Sea. Table 2 shows the individual questions. The correct answers to the multiple-choice questions are marked, and suggested solutions are given for the open questions. For the multiple-choice questions, one point was awarded for a correct answer, none for an incorrect answer. In the open questions, two components were required in each case. If both factors were named correctly, one point was awarded; if one factor was named correctly, half a point was awarded. If no correct component was presented, no point was awarded.

### 2.5. Study Design

The survey of environmental attitudes and environmental knowledge was conducted in parallel and at a total of three different time points. Specifically, students were questioned before (T0), immediately after (T1), and four to six weeks after (T2) the mudflat hiking tour. Therefore, the underlying research design of this study is a pre–post-test followed by a retention test. The same instruments presented were used at all three measurement time points. Therefore, the items were the same for all surveys.

### 2.6. Analysis

To determine the potential influence of the mudflat hiking tour on environmental attitudes and environmental knowledge about the Wadden Sea, a total of three single-factor variance analyses with repeated measures were conducted. In each case, the development of the values over the three measurement time points for preservation, utilization, and environmental knowledge was considered individually.

Various prerequisites must be met before a single-factor analysis of variance with repeated measures can be conducted. These include, among others, normal distribution, which could be assumed for all calculations due to the sufficiently large sample [58]. Another prerequisite is sphericity [59]. The performed Mauchly tests illustrate that the sphericity is neither given for preservation (Mauchly-*W* = 0.688, *p* < 0.001) nor for utilization (Mauchly-*W* = 0.809, *p* < 0.001) nor for the environmental knowledge test (Mauchly-*W* = 0.955, *p* = 0.008). Because of the magnitude of the violation of sphericity (*ε* > 0.75), a Huynh–Feldt correction was applied in each case according to recommendations from the literature [60]. Cohen’s *f* is used to indicate the magnitude of effects. An effect is small from *f* = 0.1, typical from *f* = 0.25, and large from *f* = 0.4 [61].

Pearson product–moment correlations were calculated to answer the third research question. With this method, it is possible to determine a potential correlation between the environmental attitudes and the environmental knowledge of the students. A correlation from a value of *r* = |0.1| is relatively small, from *r* = |0.2| it is typical, and from *r* = |0.3| it is described as relatively large [62].

Cronbach’s alpha was also calculated to check the internal consistency of the 2-MEV model. Table 3 shows the values for preservation and utilization determined at the three measurement points.

The values for Cronbach’s alpha presented in Table 3 demonstrate that the internal consistency of the two subscales can be rated as respectable to very good for all three measurement time points [63].

## 3. Results

The presentation of the results obtained is in the order of the three research questions set up. The first research question investigated the influence of the mudflat hiking tour on the students’ environmental attitudes. The performed one-factor variance analyses with repeated measures for preservation proves that they are statistically significantly different from each other (*F*(1.533, 329.496) = 37.652, *p* < 0.001). The Bonferroni-corrected post hoc test shows differences in the expression of preservation (T0: *M* = 35.25, *SD* = 7.25; T1: *M* = 38.62, *SD* = 5.26; T2: *M* = 38.25, *SD* = 5.67). Differences are statistically significant between measurement time points T0 and T1 and T0 and T2 (*p* < 0.001) but not between measurement time points T1 and T2 (*p* = 0.596). The effect size according to Cohen is *f* = 0.42. The one-factor variance analyses with repeated measures for utilization also prove a statistically significant difference (*F*(1.690, 363.414) = 40.903, *p* < 0.001). Using a Bonferroni-corrected post hoc test can also illustrate differences in the expression of utilization (T0: *M* = 23.48, *SD* = 6.24; T1: *M* = 20.01, *SD* = 4.89; T2: *M* = 20.69, *SD* = 4.60). Again, the difference between time points T0 and T1 and T0 and T2 is statistically significant (*p* < 0.001), and that between time points T1 and T2 is not (*p* = 0.083). The strength of the effect is *f* = 0.44.

As can be seen from the methodological section, a single-factor variance analysis with repeated measures was also performed with regard to environmental knowledge and, thus, used for the second research question. This shows a statistically significant difference (*F*(1.931, 399.623) = 265.952, *p* < 0.001). The Bonferroni-corrected post hoc test demonstrates differences in the expression of environmental knowledge (T0: *M* = 3.22, *SD* = 1.72; T1: *M* = 6.07, *SD* = 1.85; T2: *M* = 5.48, *SD* = 1.84). The differences are statistically significant at each of the three measurement time points (each *p* < 0.001). The effect size according to Cohen is *f* = 1.13. At this point, significant responses from students regarding the characteristics (Table 2, question 1) and threats to the Wadden Sea (Table 2, question 10) are given as examples. Often the Wadden Sea is described as *very large* and *accessible*. The *tides* of *ebb* and *flow* and their *effects on the living conditions* of animals and plants are also mentioned. Human influences, in particular, are pointed out as threats. In addition to various forms of *pollution, climate change, sea level rise*, and exploitation through *overfishing* are often stated.

For the third research question, a correlation analysis is used. Here, preservation and utilization were correlated with environmental knowledge at all three measurement time points. In the pretest, preservation and environmental knowledge correlated positively (*r* = 0.239, *p* < 0.001), and utilization correlated negatively with environmental knowledge (*r* = –0.164, *p* = 0.002). This pattern also emerges from the posttest data. There is a positive correlation between preservation and environmental knowledge (*r* = 0.232, *p* < 0.001), and there is a negative correlation between utilization and environmental knowledge (*r* = –0.203, *p* < 0.001). This result can be confirmed in the retention test as well. Preservation and environmental knowledge correlate positively (*r* = 0.281, *p* < 0.001), and utilization correlates negatively with environmental knowledge (*r* = –0.146, *p* = 0.017). All correlations mentioned are statistically significant.

## 4. Discussion

The results for the first research question prove the positive influence of the mudflat hiking tour on the environmental attitudes of the students. The high potential of the Wadden Sea as an out-of-school learning site for environmental education [19] can be empirically proven for the first time. Preservation, or the preference for conservation, was significantly increased in both the short term and the long term. This result is consistent with findings from another study. In this one, the influence of station work on the Wadden Sea conducted in classroom was considered, which took into account an implementation of different experiments and the use of original objects [64]. However, in empirical research of this school-based intervention, preservation decreased statistically significantly between the posttest and the retention test [65]. Although preservation scores also decreased slightly during this period in the present study, there is no significant difference between the T1 and T2 measurement time points. This is an indication of the long-term positive effect of a mudflat hiking tour. Moreover, it proves the relevance of primary experiences already presented in the school context [66,67,68].

These conclusions are also reinforced with the results related to utilization. There was a significant short-term and long-term decrease in students’ preference for nature exploitation due to the field trip to the Wadden Sea. Because there is no significant increase in this preference between time T1 and T2, the positive impact of this educational intervention at the Wadden Sea out-of-school learning site is again evident. In the case of the school-based station work on the Wadden Sea, the values for utilization between the posttest and the retention test differed significantly [65]; thus, the corresponding preference increased again. Therefore, based on the variance analyses calculated for both factors of the 2-MEV model, it can be concluded that the out-of-school mudflat hiking tour has a more long-term positive influence on the environmental attitudes of the students than a hands-on school lesson with the same content on the Wadden Sea ecosystem.

Furthermore, the results can be related to the findings obtained in relation to teaching and learning processes at other out-of-school learning sites. As shown, it is equally possible to have a positive impact on environmental attitudes with a one-day environmental education program on climate change in the botanical garden [7]. However, in this study, it was found that preservation was positively influenced only in the short term and not in the long term. Since similar results have been obtained in other contexts in the literature [46,69,70], it is even more surprising that a long-term positive influence on both factors could be made with the mudflat hiking tour. For the out-of-school learning site zoo, the importance of accompanying a visit was particularly identified, and the development of negative changes in visitors’ understanding was related to unguided visits [14]. This aspect can be taken up for the context of the mudflat hiking tour. In this way, it stands to reason that the mudflat guide made a significant contribution to the positive developments in environmental attitudes due to his explanations. In subsequent studies, this point should be investigated. Thereby, it is possible to conduct a comparative study that examines the effects of a mudflat hiking tour with and without the accompaniment of a mudflat guide.

The second research question focuses on the influence of the mudflat hiking tour on students’ environmental knowledge of this ecosystem. The statistical analysis shows positive short-term and positive long-term significant changes in environmental knowledge. So, through the mudflat hiking tour, participants learned content about this ecosystem that was presented to them not only immediately after the mudflat hiking tour, but also four to six weeks later. Although students scored significantly higher on the retention test than on the pretest, the decrease from the posttest to the retention test is equally significant. This is consistent with the results obtained from research on station work on the Wadden Sea [28]. Nonetheless, this result is positive, since even four to six weeks after the excursion a significantly higher level of environmental knowledge is present than before the mudflat hiking tour. Therefore, with this educational intervention at the out-of-school learning site, an objective of the educational concept of the national park facilities of the Lower Saxony Wadden Sea National Park could be fulfilled [35].

This result is not surprising when compared to the effects of other out-of-school learning sites. In an environmental education program in a botanical garden, both a short-term and a long-term positive impact on students’ knowledge was also found [6]. The same is found for different interventions in zoological institutions [10,11]. Ultimately, the potential of out-of-school teaching and learning processes at different out-of-school learning sites can also be confirmed on a cognitive level.

In order to relate this cognitive factor environmental knowledge to the affective factor environmental attitudes, correlations were tested by conducting correlation analyses. This has not been performed in previous studies on out-of-school learning sites, so, unfortunately, there is no possibility of an in-depth addition from the literature to discuss the results. As outlined, there is a significant positive correlation between environmental knowledge and preservation and a significant negative correlation between environmental knowledge and utilization at each of the three measurement time points. These results are not surprising and support the discussion already made regarding the first and second research questions. Students with a high preference for conservation have higher environmental knowledge of Wadden Sea than students with a lower expression of this preference. In addition, students with a high preference of nature exploitation have a lower environmental knowledge than students with a higher expression of this preference. Since these correlations could be determined completely independent of the respective measurement time points, the stability of these becomes clear. Therefore, it can be concluded that it is important to promote cognitive and affective factors in order to foster positive learning processes and environmentally friendly attitudes in learners. In subsequent studies, this aspect should be taken up, and, in parallel, the importance of other influences for this connection should be investigated. Especially because out-of-school learning is often associated with high motivation [8], the potential influence of this factor on the identified relationships could be tested. With regard to the out-of-school learning site Wadden Sea, the possible influence of disgust on cognitive and affective factors should also be examined, as it seems to be significant for the experience of this UNESCO World Heritage Site [19].

## 5. Conclusions

The present study was the first to empirically research teaching and learning processes with secondary school students at the UNESCO World Heritage Wadden Sea out-of-school learning site from an environmental education perspective. The results demonstrate a positive short-term and a positive long-term impact of the mudflat hiking tour on students’ environmental attitudes and environmental knowledge acquisition. Precisely because environmental attitudes do not differ significantly from those expressed immediately after the field trip, even four to six weeks after the educational intervention, the potential of out-of-school primary experiences of the Wadden Sea for education for sustainable development becomes clear. Therefore, the Wadden Sea as an out-of-school learning site should also be given a higher priority than before in the legal requirements for the organization of lessons, so that this potential can be increasingly used in the future and further researched in parallel. Corresponding implications were given as examples in the discussion of the results.

## Figures and Tables

**Table 1:** Items included in the 2-Major Environmental Values (2-MEV) by Bogner and Wiseman [23] on preservation vs. utilization attitudes.

Statement	Strongly Disagree	Disagree	Neutral	Agree	Strongly Agree
**Preservation**					
It upsets me to see the countryside taken over by building sites.					
I enjoy trips to the countryside.					
Humankind will die out if we don’t live in tune with nature.					
Society will continue to solve even the biggest environmental problems.					
Sitting at the edge of a pond watching dragonflies in flight is enjoyable.					
I save water by taking a shower instead of a bath (in order to spare water).					
I always switch the light off when I don’t need it.					
We must set aside areas to protect endangered species.					
It is interesting to know what kinds of creatures live in ponds or rivers.					
Dirty industrial smoke from chimneys makes me angry.					
**Utilization**					
Worrying about the environment often holds up development projects.					
We need to clear forests in order to grow crops.					
Our planet has unlimited resources.					
Nature is always able to restore itself.					
We must build more roads so people can travel to the countryside.					
Only plants and animals of economical importance need to be protected.					
Humans have the right to change nature as they see fit.					
People worry too much about pollution.					
Human beings are more important than other creatures.					
We should remove garden weeds to help beautiful flowers grow.					

**Table 2:** The environmental knowledge test on the Wadden Sea ecosystem.

**1. Describe two special characteristics of the Wadden Sea ecosystem.**	
* e.g. demanding living conditions, tidal influence, World Heritage Site*	
**2. Name two weather conditions where a mudflat hiking tour can be dangerous.**	
* e.g. thunderstorm, fog, intense heat*	
**3. In which habitat can mudgrass mainly be found?**	
o	dunes
o	mudflats
×	salt marsh
o	mudflat meadow
**4. What are the brown areas on the top of the mudflats?**	
o	dead mussels
o	excrement of birds
×	algae
o	small snails
**5. What special ability do laver spire shell have?**	
×	they can surf with the help of water
o	they can jump away from enemies
o	they can communicate with each other by smells
o	no answer is correct
**6. What are the “spaghetti piles” that can be seen all over the bottom of the Wadden Sea?**	
* e.g. piles of lugworms’ excrement, sand filtered by lugworms*	
**7. How do mussels burrow into the mudflats?**	
o	with their siphons
×	by jerky movements
o	with the help of the water
o	they can’t burrow in
**8. Name two behaviors typical of shore crabs**	
* e.g. threatening with their claws, moving sideways, digging in*	
**9. What are barnacles?**	
o	components of groynes
o	plants
o	components of the shell of shore crabs
×	crustaceans
**10. Name two threats to the Wadden Sea.**	
* e.g. global climate change, plastic waste pollution, overfishing*	

**Table 3:** The values for Cronbach’s alpha at the three measurement time points.

Subscale	T0	T1	T2
preservation	0.83	0.70	0.76
utilization	0.79	0.74	0.71

## Data Availability

The data presented in this study are available on request from the corresponding author.
